# Supplementary figures and images for: AquaX: An enhanced and revised AquaMaps framework to model marine species distributions and biodiversity
Source: PLoS One. 2026 Feb 20;21(2):e0335823. doi: 10.1371/journal.pone.0335823 (PMC12923144; doi:10.1371/journal.pone.0335823)

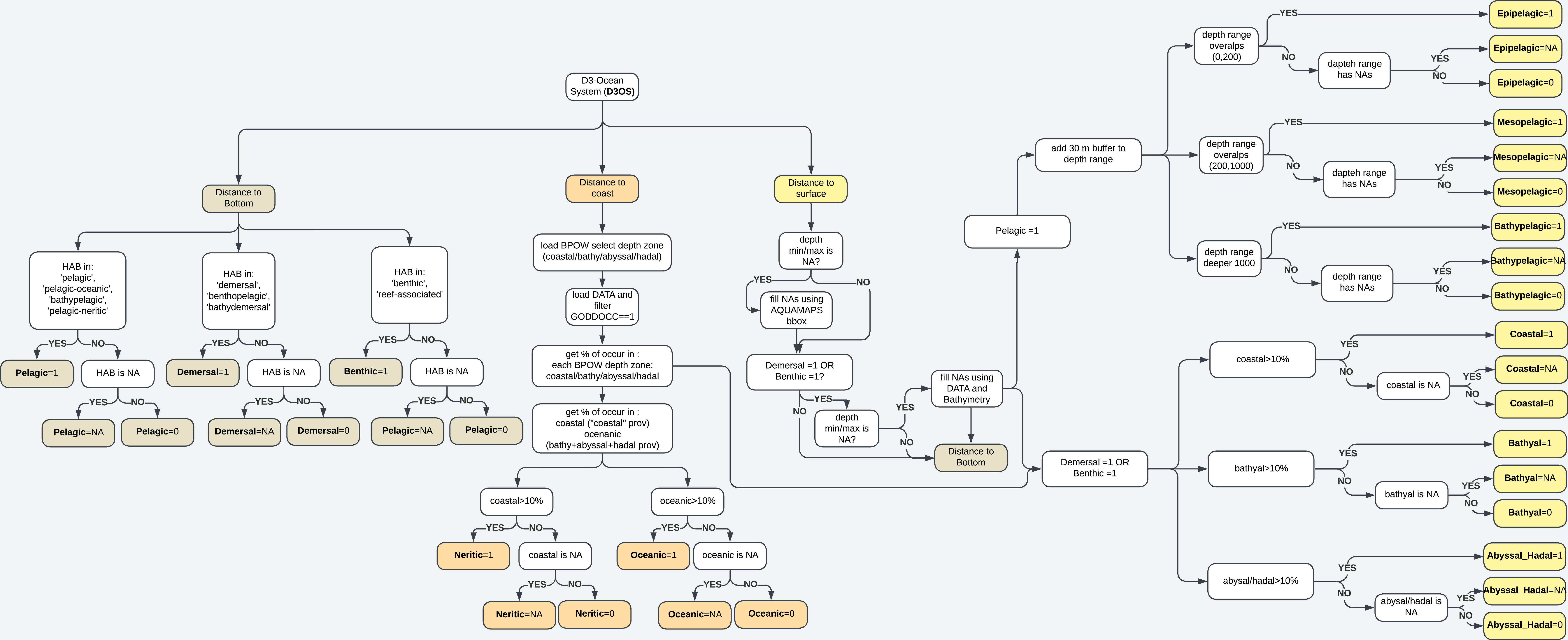

Supplement: S2 Fig — (TIF) [file pone.0335823.s002.tif]

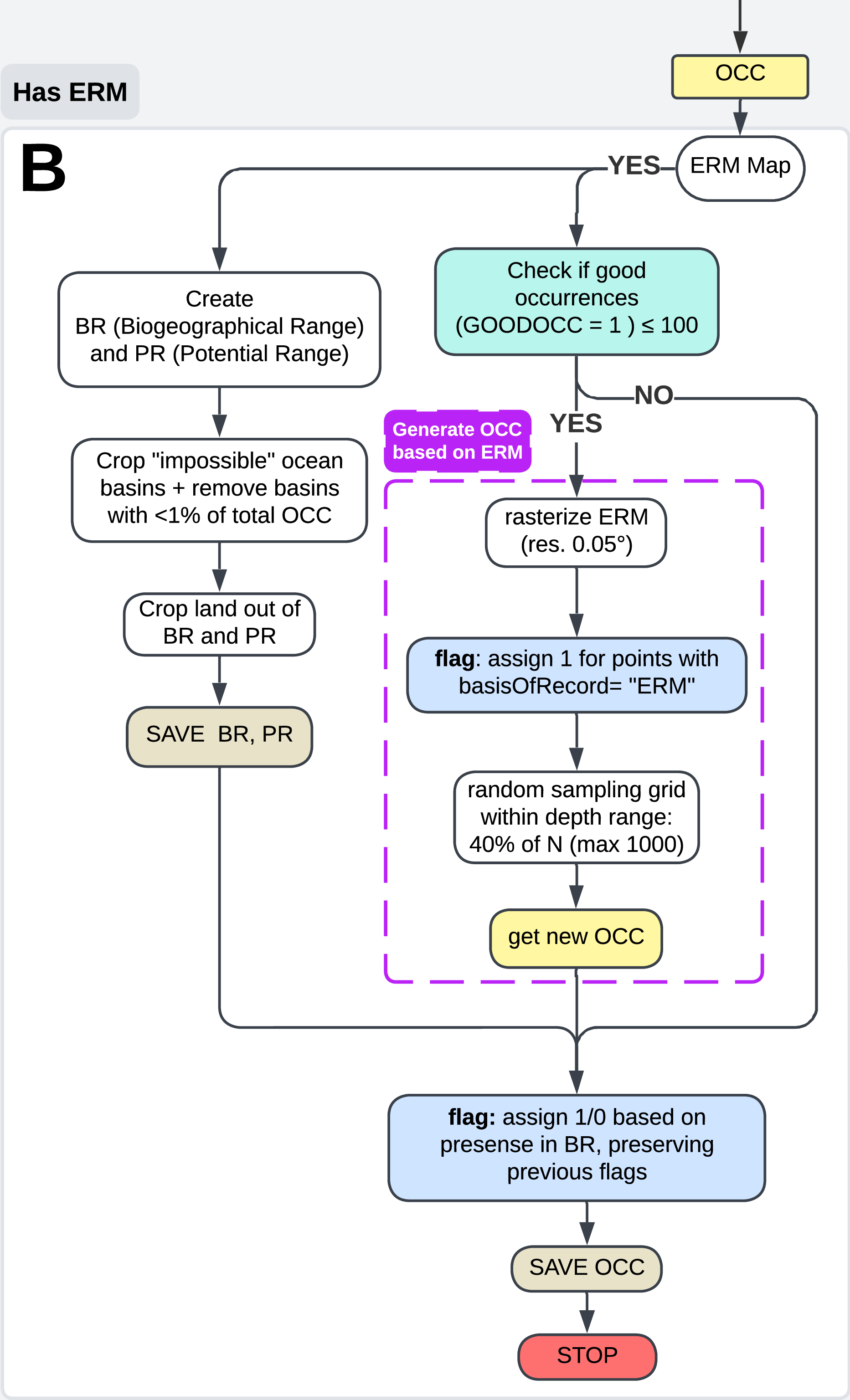

Supplement: S3 Fig — Abbreviations used: OCC – occurrence data, GOODOCC – column in OCC containing standardized binary flags, basisOfRecord – column in OCC containing occurrence type, BR – biogeographical range, PR – potential range, ERM – expert range map. (TIFF) [file pone.0335823.s003.tiff]

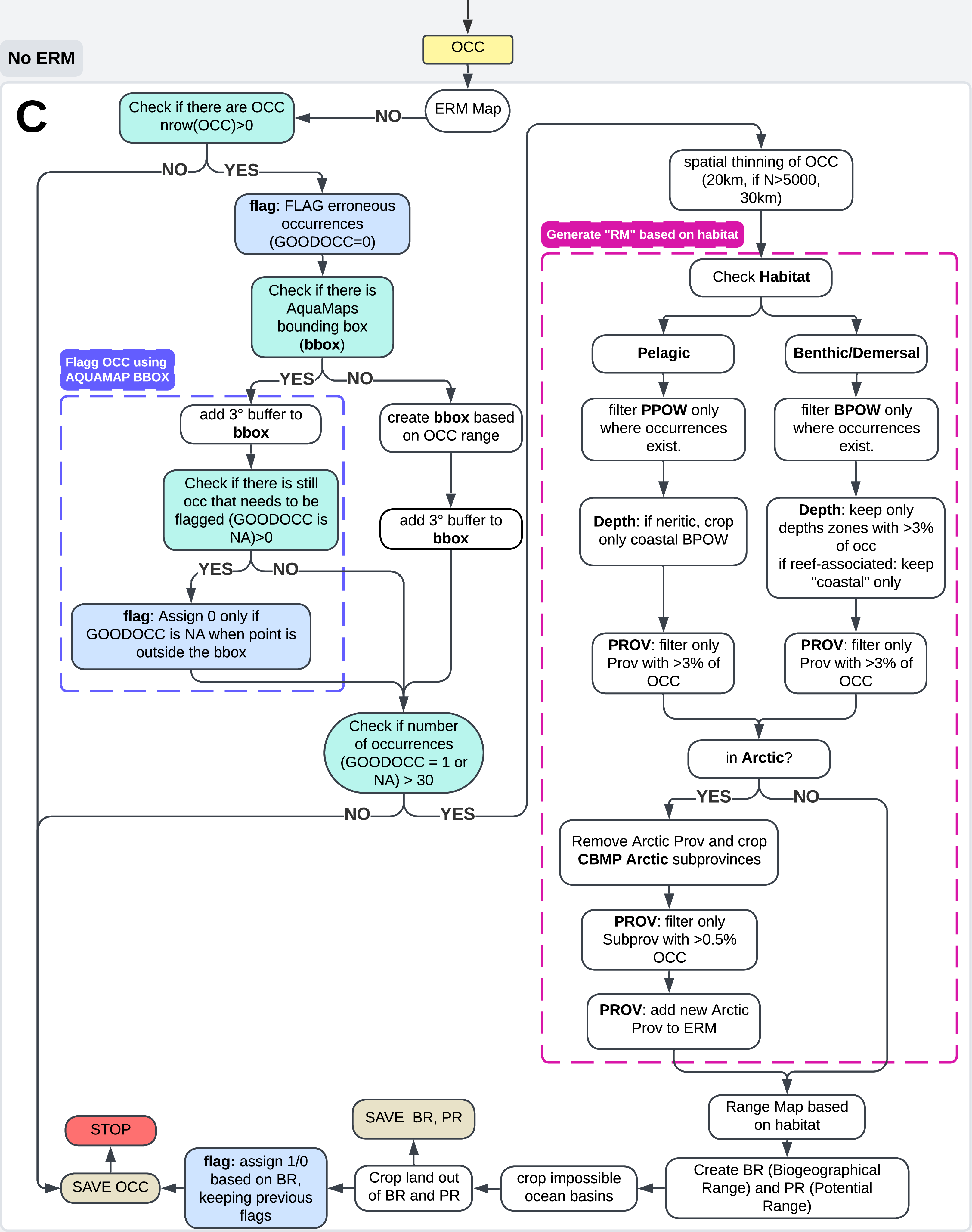

Supplement: S4 Fig — Abbreviations used: OCC – occurrence data, GOODOCC – column in OCC containing standardized binary flags, BR – biogeographical range, PR – potential range, bbox – AquaMaps bounding box, Prov – Province, ERM – expert range map, N – number of observations in OCC. (TIF) [file pone.0335823.s004.tif]

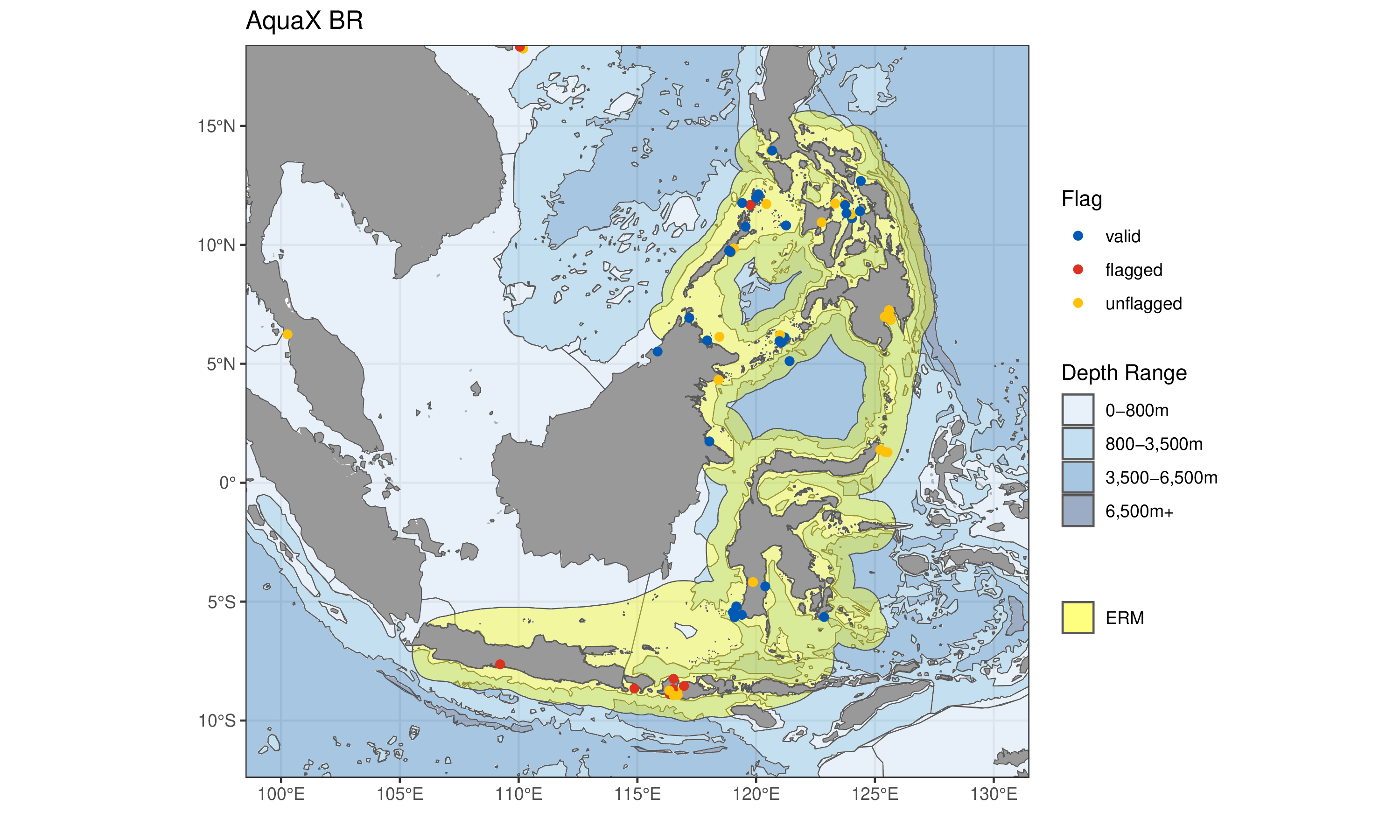

Supplement: S9 Fig — Erroneous (flagged) occurrences are marked by red, verified (valid) occurrence points are marked in blue, records with no flags (unflagged) are marked as yellow. Depth ranges from BPOW are shaded in blues. Made with Natural Earth. Free vector and raster map data @ naturalearthdata.com. (TIFF) [file pone.0335823.s009.tiff]

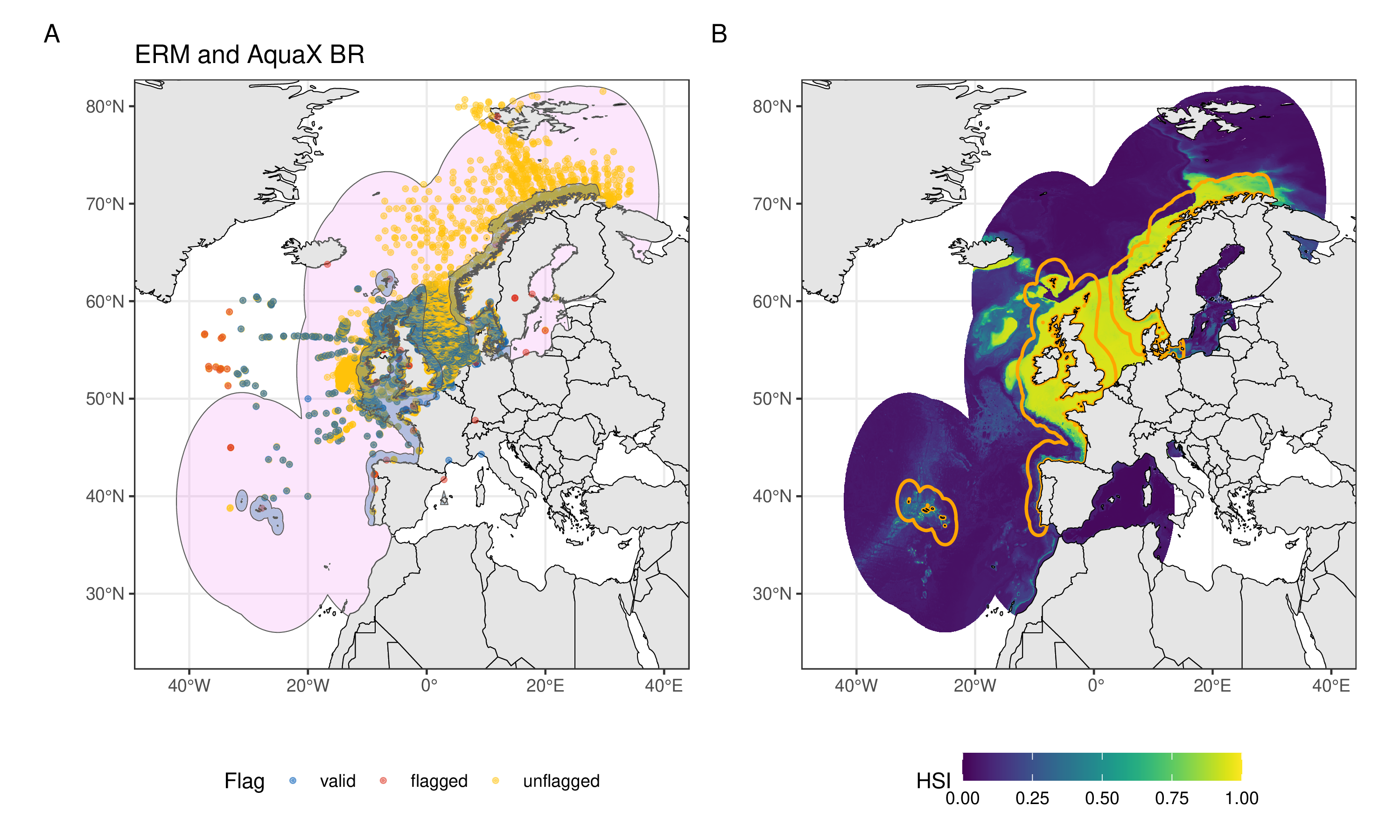

Supplement: S10 Fig — A) Expert range map (shaded blue), Potential Range (shaded light violet) and raw occurrence data of snake pipefish. Erroneous (flagged) occurrences are marked by red, verified (valid) occurrence points are marked in blue, records with no flags (unflagged) are marked as yellow. B) Ensemble mean habitat suitability index (HSI) of snake pipefish. Orange line is the outline of the expert range map (IUCN ERM). Made with Natural Earth. Free vector and raster map data @ naturalearthdata.com. (TIFF) [file pone.0335823.s010.tiff]

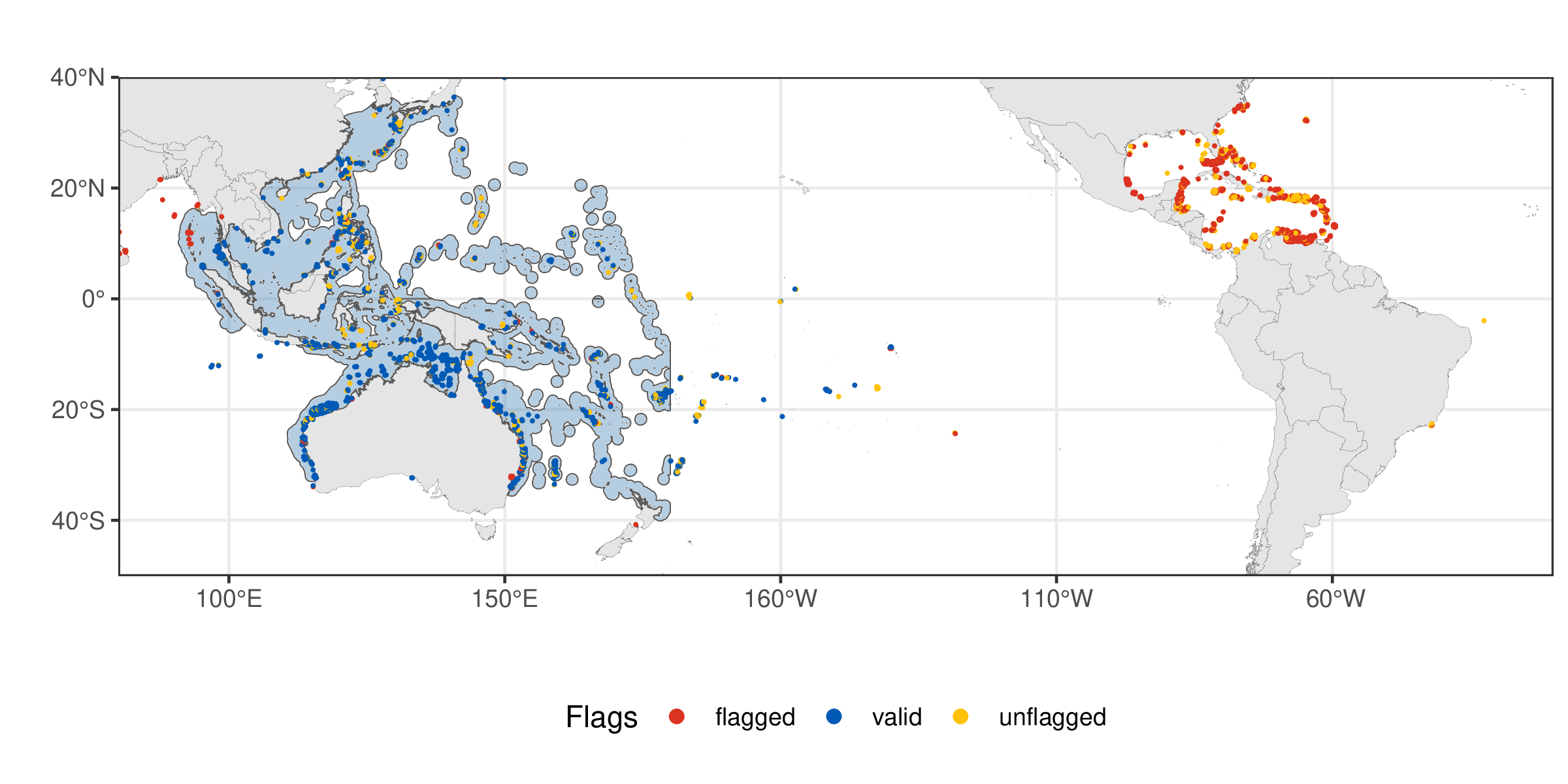

Supplement: S11 Fig — Erroneous (flagged) occurrences are marked by red, verified (valid) occurrence points are marked in blue, records with no flags are marked as yellow. Made with Natural Earth. Free vector and raster map data @ naturalearthdata.com. (TIFF) [file pone.0335823.s011.tiff]
