# Supplementary material for: AquaX: An enhanced and revised AquaMaps framework to model marine species distributions and biodiversity
Source: PLoS One. 2026 Feb 20;21(2):e0335823. doi: 10.1371/journal.pone.0335823 (PMC12923144; doi:10.1371/journal.pone.0335823)
Supplement: Attachment — Submitted filename: review_PLoSONE_AquaX.pdf [file pone.0335823.s012.pdf]

They study by Reygondeau et al. titled “AquaX: An enhanced and revised AquaMaps framework to model marine species distributions and biodiversity” presents the upgraded version of AquaMaps, a valuable tool that models and provides global maps of current and future/potential species distributions. The manuscript is generally very well written and thoroughly describes the methodology using high quality, informative figures. I particularly appreciate the extensive, detailed, and honest section on the multi-leveled limitations of the method. Despite those, AquaX remains a valuable tool that is expected to be extensively used globally. I suggest minor revisions and provide specific comments below. One important comment has to do with polygon buffering. Other comments focus on enhancing certain discussion parts with relevant references. Because the results are (understandably) presented together with the discussion, sometimes the discussion portion lacks broader context through citations.

Line 55 “ocean”: perhaps "marine" would be more suitable here, as more general and inclusive. in this case, I would suggest the following structure: ...including ecosystem complexity and difficulty of sampling the marine realm.

Line 57 “Ecological Niche”: why are these capitalized?

Line 60 “develop”: developed

Line 76 “introduction”: this section could benefit from some more references regarding SDMs and more.

suggested refs:

https://www.frontiersin.org/journals/marine-science/articles/10.3389/fmars.2017.00421/full

https://nsojournals.onlinelibrary.wiley.com/doi/full/10.1002/ecog.07702

https://onlinelibrary.wiley.com/doi/full/10.1111/gcb.16371

line 79 “SDG”: please spell out the full word for this first mention

line 128 “also strengthens”: suggestion: would also strengthen

line 129 “makes”: make

line 132: delete “and”; comma after “algorithms

line 143 “enable”: enables

line 148 “evidences”: evidence

line 151 “explore”: is it to explore or really to present?

Line 165 “AphiaID”: please add this “Aphia unique identifiers” since this is the first mention

Line 195 “GOODOCC”: I don't think this has been previously defined in the text

Line 213 “FishBase”: add “for fishes”

Line 214 “SeaLifeBase”: add “for other marine organisms”

Line 217 “describe precisely”: precisely describe

Line 240 “varies here from”: varies based on? or differs from? which of the two?

Line 278 “buffering of 10o of longitude and latitude”: isn't this huge? Ten degrees of latitude ≈ 1,110 km. so even for 100 years, this would be around 5 degrees change. also, a poleward shift is by definition directional, while buffering expands in all directions.

Line 368 “(2000-2010) and two future periods (2050-”: what about 2011-2049?

Line 413 “unique environments”: what is the purpose of this process?

Line 444 “models algorithm”: Model algorithms?

Line 508-9 “three environmental parameters available at the time”: which ones? it would be useful to mention in a parenthesis

Line 515 “through”: the new AquaX framework

Line 523 “replicable on”: in

Line 537-40: add one or two relevant references

Line 550 “frame”: frames?

Line 554: in “the” FISH-MIP and CMIP projects

Line 585: A tool for expert-based spatial delineation of species ranges?

Line 588: perhaps after “AquaMaps” add something along the lines of ", the basis of AquaX,"

Line 596-600: relevant references that express such needs could be cited here

Line 609 “to continue to”: add “further”

Line 614 “actions”: reference? most statements in this part could be supported by relevant refs. this section is still discussion, not just results

Line 619 “analysis”: analyses

Line 624 “The new”: it would be more intuitive if you presented the results in this section first and then have the discussion part, which is the first paragraph, to my understanding

Line 624-5 “which presented with different ecology”: this phrasing is confusing

Line 626 “the outputs”: delete “the”

Line 628: “the” ERM

Line 629 “extended”: “extends”

Line 635 “habitat modifications”: what is meant by that?

Line 640 “RCP”: was this defined before in the text?

Line 642-4: references for this statement?

Line 646 “species”: reference for this statement?

Line 648 “For example”: suggested: For example, in the case of sand trout, an increase in HSI is projected in its northward range by mid-century (Fig. 3E–F), along with a potential shift to deeper waters (blue areas below the red strip in Fig. 3F).

Line 649 “mid of the century”: mid-century

Line 654 “compute to”: should this be deleted?

Line 658 “providing”: this sentence is not finished

Line 666: Furthermore, the AquaX-derived species richness …

Line 668 “resampled”: resampling?

Line 669 “using”: replace with “with” or “employing”

Line 670 “dataset”: datasets

Line 687 “approach”: I would delete this

Line 706 “depth ranges”: I wonder if depth data from surveys have been or can be integrated. suggested source to explore: https://james-thorson.github.io

Line 720 “with a depth range from 0-12m”: based on what?

Line 747: records, with most …

Line 772: verifications), and 5.4%

Line 805: any relevant refs?

Line 807-8 “and attempt to follow update on ERM at a by-annually level”: this sentence needs rephrasing

Line 834: high-sea

Line 857-9: so, it affects the SSP projections here as well which is quite important

Line 862: this seems to be addressed in a previous section - why not combine the two? a couple of references would be useful for readers here

Line 872 “unabling”: hindering .. from making?

Line 908 “disk”: was this explained previously? perhaps a short explanation would still help here

Line 921 “bottom trawling”: fishing was included in this paper, perhaps it could be cited

https://www.pnas.org/action/oidcCallback?idpCode=connect&error=login_required&error_description=Login+required&state=Dps2IO0LOrpSUAYYguc7KjWtugvQmVzet9SRzoptjvlX2D8yKiJIuCAF5rn25evTKSYeBJHZxgNLEKhORjMUMTOXbOnAuqUcDJMGMt4%2BX7v0loW6TTUMWc%2BXGrsT9EEsIXiBtzIJTsTd4F0rE1Tm6w%3D%3D

line 928: a reference or two?

Line 1228: which color represents what (invasion vs extinction)? there is no color scale

Line 1229: the middle … and end … of the century

Line 1233-4 “lost or gained”: which color represents which?

Line 1270: my comments on this Fig. are resolved when reading the text, but I believe these details should be added to the fig. legend for clarity

Line 1272 “flags” and “occurrence type”: what kind of flags? And consider adding two examples. (e.g., xxx and xxx).

Line 1287 “ CSI, AUC and TSS”: these should be defined in the legend so it is stand-alone

Line 1288 “300 runs. Boxplot for each single models are shown as well as outliers (point).”: please rephrase for better flow and typos

Line 1289: what does this mean "DepthRangeShallow_NA"? that there are 6000 missing values of what? of shallow species? the NA at the end is also confusing

Line 1301-2: italicize the species

Fig. S6: x axis label typo “validation”
