# Supplementary material for: AquaX: An enhanced and revised AquaMaps framework to model marine species distributions and biodiversity
Source: PLoS One. 2026 Feb 20;21(2):e0335823. doi: 10.1371/journal.pone.0335823 (PMC12923144; doi:10.1371/journal.pone.0335823)
Supplement: Attachment — Submitted filename: reviewer_comments_PLoSONE_AquaX.docx [file pone.0335823.s014.docx]

# Editors’ Comments

### When submitting your revision, we need you to address these additional requirements.

### 1. Please ensure that your manuscript meets PLOS ONE's style requirements, including those for file naming. The PLOS ONE style templates can be found at

### [https://journals.plos.org/plosone/s/file?id=wjVg/PLOSOne_formatting_sample_main_body.pdf](https://urldefense.com/v3/__https:/journals.plos.org/plosone/s/file?id=wjVg*PLOSOne_formatting_sample_main_body.pdf__;Lw!!KVu0SnhVq1hAFvslES2Y!N2B9KAiF0TPDAaxnezlRYssg9gxj1OwjaijSvFybGFHCQExKRGeNqm2Q0RaworCLbbhuUr7Qa3xJ-OIOh_qzk9m6GW46Majv$) and

### [https://journals.plos.org/plosone/s/file?id=ba62/PLOSOne_formatting_sample_title_authors_affiliations.pdf](https://urldefense.com/v3/__https:/track.editorialmanager.com/CL0/https:*2F*2Fjournals.plos.org*2Fplosone*2Fs*2Ffile*3Fid=ba62*2FPLOSOne_formatting_sample_title_authors_affiliations.pdf/1/010f019a70b3590e-dd193eae-5cb3-4253-b76c-32b77b17b98a-000000/1CpYgSLucj0u2znYgtNrj3OdWmho1LH5qoZfAYrEhNg=236__;JSUlJSUlJQ!!KVu0SnhVq1hAFvslES2Y!N2B9KAiF0TPDAaxnezlRYssg9gxj1OwjaijSvFybGFHCQExKRGeNqm2Q0RaworCLbbhuUr7Qa3xJ-OIOh_qzk9m6Genq9O6A$)

**Answer**: The content of the revised manuscript and file names has been adjusted to meet the journal style requirements

### 2. PLOS requires an ORCID iD for the corresponding author in Editorial Manager on papers submitted after December 6th, 2016. Please ensure that you have an ORCID iD and that it is validated in Editorial Manager. To do this, go to ‘Update my Information’ (in the upper left-hand corner of the main menu), and click on the Fetch/Validate link next to the ORCID field. This will take you to the ORCID site and allow you to create a new iD or authenticate a pre-existing iD in Editorial Manager.

**Answer**: The ORCID iD is added to the corresponding author account.

### 3. Thank you for uploading your study's underlying data set. Unfortunately, the repository you have noted in your Data Availability statement does not qualify as an acceptable data repository according to PLOS's standards.

### At this time, please upload the minimal data set necessary to replicate your study's findings to a stable, public repository (such as figshare or Dryad) and provide us with the relevant URLs, DOIs, or accession numbers that may be used to access these data. For a list of recommended repositories and additional information on PLOS standards for data deposition, please see [https://journals.plos.org/plosone/s/recommended-repositories](https://urldefense.com/v3/__https:/track.editorialmanager.com/CL0/https:*2F*2Fjournals.plos.org*2Fplosone*2Fs*2Frecommended-repositories/1/010f019a70b3590e-dd193eae-5cb3-4253-b76c-32b77b17b98a-000000/b-28V8LEqAR_pwmpf20yrHmrL1gBz4pBMEoQNgyLyhM=236__;JSUlJSU!!KVu0SnhVq1hAFvslES2Y!N2B9KAiF0TPDAaxnezlRYssg9gxj1OwjaijSvFybGFHCQExKRGeNqm2Q0RaworCLbbhuUr7Qa3xJ-OIOh_qzk9m6Gd0xP68f$).

**Answer**: Manuscript data has been deposit to Figshare DOI: 10.6084/m9.figshare.30754358

### 4. We note that Figure 2, 3, 4, 5, 6, 7, S9, S10, and S11 in your submission contain map images which may be copyrighted. All PLOS content is published under the Creative Commons Attribution License (CC BY 4.0), which means that the manuscript, images, and Supporting Information files will be freely available online, and any third party is permitted to access, download, copy, distribute, and use these materials in any way, even commercially, with proper attribution. For these reasons, we cannot publish previously copyrighted maps or satellite images created using proprietary data, such as Google software (Google Maps, Street View, and Earth). For more information, see our copyright guidelines: [http://journals.plos.org/plosone/s/licenses-and-copyright](https://urldefense.com/v3/__http:/journals.plos.org/plosone/s/licenses-and-copyright__;!!KVu0SnhVq1hAFvslES2Y!N2B9KAiF0TPDAaxnezlRYssg9gxj1OwjaijSvFybGFHCQExKRGeNqm2Q0RaworCLbbhuUr7Qa3xJ-OIOh_qzk9m6GZrtgcNA$).

### We require you to either (1) present written permission from the copyright holder to publish these figures specifically under the CC BY 4.0 license, or (2) remove the figures from your submission:

### 1). You may seek permission from the original copyright holder of Figure 2, 3, 4, 5, 6, 7, S9, S10 and S11 to publish the content specifically under the CC BY 4.0 license.

### We recommend that you contact the original copyright holder with the Content Permission Form ([http://journals.plos.org/plosone/s/file?id=7c09/content-permission-form.pdf](https://urldefense.com/v3/__http:/journals.plos.org/plosone/s/file?id=7c09*content-permission-form.pdf__;Lw!!KVu0SnhVq1hAFvslES2Y!N2B9KAiF0TPDAaxnezlRYssg9gxj1OwjaijSvFybGFHCQExKRGeNqm2Q0RaworCLbbhuUr7Qa3xJ-OIOh_qzk9m6GTRqCooF$)) and the following text:

### “I request permission for the open-access journal PLOS ONE to publish XXX under the Creative Commons Attribution License (CCAL) CC BY 4.0 ([http://creativecommons.org/licenses/by/4.0/](https://urldefense.com/v3/__http:/creativecommons.org/licenses/by/4.0/__;!!KVu0SnhVq1hAFvslES2Y!N2B9KAiF0TPDAaxnezlRYssg9gxj1OwjaijSvFybGFHCQExKRGeNqm2Q0RaworCLbbhuUr7Qa3xJ-OIOh_qzk9m6GQGRdfXP$)). Please be aware that this license allows unrestricted use and distribution, even commercially, by third parties. Please reply and provide explicit written permission to publish XXX under a CC BY license and complete the attached form.”

### Please upload the completed Content Permission Form or other proof of granted permissions as an "Other" file with your submission.

### In the figure caption of the copyrighted figure, please include the following text: “Reprinted from [ref] under a CC BY license, with permission from [name of publisher], original copyright [original copyright year].”

### 2).If you are unable to obtain permission from the original copyright holder to publish these figures under the CC BY 4.0 license or if the copyright holder’s requirements are incompatible with the CC BY 4.0 license, please either i) remove the figure or ii) supply a replacement figure that complies with the CC BY 4.0 license. Please check copyright information on all replacement figures and update the figure caption with source information. If applicable, please specify in the figure caption text when a figure is similar but not identical to the original image and is therefore for illustrative purposes only.

### The following resources for replacing copyrighted map figures may be helpful:

### USGS National Map Viewer (public domain): [http://viewer.nationalmap.gov/viewer/](https://urldefense.com/v3/__http:/viewer.nationalmap.gov/viewer/__;!!KVu0SnhVq1hAFvslES2Y!N2B9KAiF0TPDAaxnezlRYssg9gxj1OwjaijSvFybGFHCQExKRGeNqm2Q0RaworCLbbhuUr7Qa3xJ-OIOh_qzk9m6GfdZDIi5$)

### The Gateway to Astronaut Photography of Earth (public domain): [http://eol.jsc.nasa.gov/sseop/clickmap/](https://urldefense.com/v3/__http:/eol.jsc.nasa.gov/sseop/clickmap/__;!!KVu0SnhVq1hAFvslES2Y!N2B9KAiF0TPDAaxnezlRYssg9gxj1OwjaijSvFybGFHCQExKRGeNqm2Q0RaworCLbbhuUr7Qa3xJ-OIOh_qzk9m6GdVbAYqi$)

### Maps at the CIA (public domain): [https://www.cia.gov/library/publications/the-world-factbook/index.html](https://urldefense.com/v3/__https:/www.cia.gov/library/publications/the-world-factbook/index.html__;!!KVu0SnhVq1hAFvslES2Y!N2B9KAiF0TPDAaxnezlRYssg9gxj1OwjaijSvFybGFHCQExKRGeNqm2Q0RaworCLbbhuUr7Qa3xJ-OIOh_qzk9m6GSPe8qDN$) and [https://www.cia.gov/library/publications/cia-maps-publications/index.html](https://urldefense.com/v3/__https:/www.cia.gov/library/publications/cia-maps-publications/index.html__;!!KVu0SnhVq1hAFvslES2Y!N2B9KAiF0TPDAaxnezlRYssg9gxj1OwjaijSvFybGFHCQExKRGeNqm2Q0RaworCLbbhuUr7Qa3xJ-OIOh_qzk9m6GVGYEZJ8$)

### NASA Earth Observatory (public domain): [http://earthobservatory.nasa.gov/](https://urldefense.com/v3/__http:/earthobservatory.nasa.gov/__;!!KVu0SnhVq1hAFvslES2Y!N2B9KAiF0TPDAaxnezlRYssg9gxj1OwjaijSvFybGFHCQExKRGeNqm2Q0RaworCLbbhuUr7Qa3xJ-OIOh_qzk9m6GVbZt6qx$)

### Landsat: [http://landsat.visibleearth.nasa.gov/](https://urldefense.com/v3/__http:/landsat.visibleearth.nasa.gov/__;!!KVu0SnhVq1hAFvslES2Y!N2B9KAiF0TPDAaxnezlRYssg9gxj1OwjaijSvFybGFHCQExKRGeNqm2Q0RaworCLbbhuUr7Qa3xJ-OIOh_qzk9m6GeyGrMNS$)

### USGS EROS (Earth Resources Observatory and Science (EROS) Center) (public domain): [http://eros.usgs.gov/#](https://urldefense.com/v3/__http:/eros.usgs.gov/*__;Iw!!KVu0SnhVq1hAFvslES2Y!N2B9KAiF0TPDAaxnezlRYssg9gxj1OwjaijSvFybGFHCQExKRGeNqm2Q0RaworCLbbhuUr7Qa3xJ-OIOh_qzk9m6GQGsXXzN$)

### Natural Earth (public domain): [http://www.naturalearthdata.com/](https://urldefense.com/v3/__http:/track.editorialmanager.com/CL0/http:*2F*2Fwww.naturalearthdata.com*2F/1/010f019a70b3590e-dd193eae-5cb3-4253-b76c-32b77b17b98a-000000/kWx1Rab1z4H_gP0mo_8UKAGcu-31bvLyxj4AhH4Gebs=236__;JSUl!!KVu0SnhVq1hAFvslES2Y!N2B9KAiF0TPDAaxnezlRYssg9gxj1OwjaijSvFybGFHCQExKRGeNqm2Q0RaworCLbbhuUr7Qa3xJ-OIOh_qzk9m6GRSOPniU$)

**Answer**: all maps were produced using tmap package that utilized natural Earth data (public domain). Each figure caption that show map now contains the following attribution: Map data were obtained from Natural Earth (public domain; naturalearthdata.com). Attributions were also given to any images that use animal silhouettes.

### 5. Please remove your figures from within your manuscript file, leaving only the individual TIFF/EPS image files, uploaded separately. These will be automatically included in the reviewers’ PDF.

**Answer**: All figures have been removed from the manuscript file. Only the figure captions remain in the manuscript, placed in the correct locations according to PLOS guidelines. All corresponding TIFF image files have been uploaded separately as individual figure files. We confirm that no figures are embedded in the manuscript, and the submission now conforms fully to the PLOS figure-upload requirements.

### 6. We are unable to open your Supporting Information file “manuscript_script.Rmd.zip” Please kindly revise as necessary and re-upload.

**Answer**: manuscript_script.Rmd.zip was autogenerated during submission process and us erroneous. he full analysis script (manuscript_script.Rmd) used to generate the results and figures has been deposited on Figshare together with the dataset and is accessible at https://doi.org/10.6084/m9.figshare.30754358

### 7. We notice that your supplementary figures are included in the manuscript file. Please remove them and upload them with the file type 'Supporting Information'. Please ensure that each Supporting Information file has a legend listed in the manuscript after the references list.

**Answer**: We notice that your supplementary figures are included in the manuscript file. Please remove them and upload them with the file type 'Supporting Information'. Please ensure that each Supporting Information file has a legend listed in the manuscript after the references list.

### 8. If the reviewer comments include a recommendation to cite specific previously published works, please review and evaluate these publications to determine whether they are relevant and should be cited. There is no requirement to cite these works unless the editor has indicated otherwise.

**Answer**: we have followed reviewer suggestion to add more references to support our claims

### 9. Please review your reference list to ensure that it is complete and correct. If you have cited papers that have been retracted, please include the rationale for doing so in the manuscript text, or remove these references and replace them with relevant current references. Any changes to the reference list should be mentioned in the rebuttal letter that accompanies your revised manuscript. If you need to cite a retracted article, indicate the article’s retracted status in the References list and also include a citation and full reference for the retraction notice.

**Answer**: We have thoroughly reviewed our reference list for completeness, accuracy, and retracted publications. During this process, we (i) removed references that are no longer cited in the revised manuscript, (ii) updated and corrected citation details where needed, and (iii) added several recent and relevant studies to better reflect the current state of the field. We have replaced the original reference list with a new, streamlined list in the revised manuscript, which now complies with the PLOS ONE reference and citation style. We checked all cited journal articles and reports against the publisher websites and major bibliographic databases and, to the best of our knowledge, none of the references currently cited in the manuscript have been retracted. Accordingly, no retraction notices needed to be added and no rationale for citing retracted work was required.

# Reviewer #1:

### This paper describes the next-generation version of the AquaMaps Ecological Niche Model, named AquaX, intended as an operational methodology that can, in theory, be adapted to many species and environmental data after addressing the challenges associated with integrating multi-source data. Due to its combination of simplicity and effectiveness, AquaMaps has served as a reference and the primary guide for approximately assessing the presence of many species, including rare ones. I recognize that proposing a new alternative model capable of producing detailed maps for over 30,000 species is ambitious; however, it holds significant potential for global conservation and planning. Therefore, this paper has great potential to become a reference in the field. Generally, the paper is sound and comprehensive. The Introduction effectively explains the context and objectives of the study. The procedure for assessing occurrence quality greatly extends that of the previous model. The innovative management of ecological and biogeographical information is both reasonable and intriguing. The implementation of range maps is also interesting and replicable. I especially appreciated the multi-scale approach and differentiation by depth layer. Overall, the methodology exemplifies an integrative system that leverages publicly available knowledge and repositories, along with a novel modelling approach well distinct from AquaMaps. The paper reflects the authors' extensive effort, with a thorough discussion and a results section that explores various applications of the methodology.

**Answer**: we would like to thank the reviewer for positive feedback and for valuable comments thought the manuscript


Specific Comments:

### + Lines 194-195: Is the GOODOCC set to 1 in all other cases?

**Answer**: in this sentence we were talking about OBIS original flags ( as shown in diagram S1_Fig1)

There is no single standardized set of flags as different databases flag tehir datat differently. That is why we have 2 sets of flag columns: 1) the original data-source flags (original_flags) and 2) the standardized binary flags (GOODOCC). The content of original_flags depends on the provider. For example, GBIF does not supply native flags, so this column remains empty, as shown in the diagram. In contrast, OBIS provides extensive data-quality flags (see OBIS Manual: “18 Data quality flags”, https://manual.obis.org/dataquality.html), and these are recorded in original_flags. As indicated in the diagram, occurrences are assigned a standardized flag value of 0 only when the original OBIS flags indicate that the point falls on land, has coordinates of (0,0), or contains other latitude/longitude errors. The rest of the flags are ignored (not flagged), and their verifications is performed later in the process (S1 Figs 3-4). AquaMaps uses a binary flagging system in which 1 indicates a good occurrence and 0 indicates a bad occurrence, as described in the manuscript. For this reason, all flags are standardized to a binary format and stored in the GOODOCC column. The data-cleaning steps (ie further assigning flags for occurrence points that do not have flags) then continue as outlined in subsequent diagrams.

We re-wrote the sentence in the following way: Other original OBIS flags, such as missing depth information or occurrences falling outside known bathymetric limits, were retained in original flag column but not automatically considered erroneous, and their GOODOCC value was left blank in standardized binary flag column (GOODOCC).

### + Line 201: Is NA equivalent to the "blank" value you assigned for OBIS? Could you standardize the notation, please?

**Answer**: thank you for pointing out this inconsistence. The sentence is re-written as following: “Since GBIF does not include explicit quality flags in its dataset, and only records without spatial issues and with valid coordinates were downloaded, the flag column for all GBIF entries was left blank to reflect the absence of a source-provided quality classification.”

### + Lines 206-210: I do not understand how GBIF is utilized in this process.

**Answer**: to illustrate the process : below you can find the hypothetical example of the duplicated data: we consider this entries duplicated because the coordinates and time of collection is identical in this case. One records come from AquaMaps database, the other comes form GBIF. The only difference here that this record was validated by AquaMaps team and flagged as good ( GOODOCC column =1) while GBIF has unflagged data (NA in GOODOCC). Hence in order not to store the duplicated information, we decided to keep the most complete one: in this case is the occurrence record from AquaMaps, as GBIF record does not contribute any extra information here.

| **latitude** | **longitude** | **dd/mm/yyyy** | **DataBase** | **GOODOCC** | AphiaID |
| --- | --- | --- | --- | --- | --- |
| 10 | 10 | 10/10/2010 | AquaMaps | 1 | 111111 |
| 10 | 10 | 10/10/2010 | GBIF | NA | 111111 |

### + Lines 300-304: How are blank and NA occurrences handled in this process?

**Answer**: We remove only records explicitly flagged as bad (GODOCC = 0). When the flag is blank, we cannot determine whether the record is good or bad; therefore, for defining the bounding box we retain all available records, including those flagged as 1 or left blank.

### + Lines 415-418: This appears to be traditional clustering. How did you identify the distinct environmental combinations?

**Answer**: To identify distinct environmental combinations, each environmental variable was first rounded to predefined, ecologically meaningful increments (Table 2). Rounding converts continuous variables into discrete bins; each bin corresponds to a single category for that variable. Unique environments were then defined as every unique combination of these binned values across all variables. This produces a set of discrete, multidimensional environmental categories rather than clusters generated by an algorithm. Each occurrence record was assigned to one such category, and one record was randomly retained per category to ensure balanced environmental representation.

### + Line 445: Please remove the initial "(".

**Answer**: The issue has been addressed in accordance with the reviewer’s suggestion.

### + Lines 445-453: Please specify why you selected these models over others or a subset. Many methods overlap in their foundational assumptions, particularly in the partitioning of the feature space during modelling (e.g., tree-based algorithms). Only a few methods, such as neural networks, can model complex relationships between data. It is possible that different models may exhibit very similar behaviour, leading the ensemble assessments to reflect less complex inter-data relationships.

**Answer**: the matter is discussed in Results and discussion section (final major update in the subsection on the evolution of AquaMaps methodology):

“The final major update of AquaX employs an ensemble modeling approach comprising ten independent models, inspired by methodologies utilized in the FISH-MIP and CMIP projects [56,57]. Each model was specifically selected to encompass various types of ENM and species distribution modeling (SDM) techniques, including linear models (GLM, GAM, MARS), background or entropy-based models (MAXNET), explorative statistical approaches (CTA, RF, FDA), and machine learning models featuring pruning and boosting techniques (ANN, GBM, XGBOOST). This combination of diverse modeling approaches aligns with best practices recommended in terrestrial ecology [39,58,59] to effectively capture the multidimensional complexity inherent in ecological niches. The ensemble output, despite potentially overestimating niche boundaries and underestimating central niche probabilities due to averaging effects [60], provides an optimal compromise, especially when projecting under future environmental scenarios.”

### + Line 628: Shouldn't it be "(Ginsburg, 1930)", with parentheses?

**Answer**: We thought so too! But we have been corrected by our co-author who is a taxonomic expert who manually reviewed the nomenclature and author citations. The use of “Ginsburg, 1930” without parentheses is correct for *Cynoscion arenarius* because the species was originally described in the same genus in which it is currently placed. Parentheses are used only when a species has been moved to a different genus than the one in which it was originally described. To ensure accuracy across all taxa, the full set of scientific names and authorships used in AquaX was verified following the World Register of Marine Species (WoRMS) conventions.

You can check the absence of parathesis for *Cynoscion arenarius* in here <https://www.marinespecies.org/aphia.php?p=taxdetails&id=276077> and for example use of parenthesis for *Thunnus albacares* here <https://www.marinespecies.org/aphia.php?p=taxdetails&id=127027>

### + Line 692: "Information" should be written in lowercase.

**Answer**: The issue has been addressed in accordance with the reviewer’s suggestion.

# Reviewer #2

### The study by Reygondeau et al. titled “AquaX: An enhanced and revised AquaMaps framework to model marine species distributions and biodiversity” presents the upgraded version of AquaMaps, a valuable tool that models and provides global maps of current and future/potential species distributions. The manuscript is generally very well written and thoroughly describes the methodology using high quality, informative figures. I particularly appreciate the extensive, detailed, and honest section on the multi-leveled limitations of the method. Despite those, AquaX remains a valuable tool that is expected to be extensively used globally. I suggest minor revisions and provide specific comments below. One important comment has to do with polygon buffering. Other comments focus on enhancing certain discussion parts with relevant references. Because the results are (understandably) presented together with the discussion, sometimes the discussion portion lacks broader context through citations.

**Answer**: We thank the reviewer for the positive and constructive assessment of our manuscript. We appreciate the recognition of the methodological improvements, and the detailed evaluation of the framework’s limitations. All comments have been carefully considered, and line-by-line responses and corresponding revisions are provided below.

### +Line 55 “ocean”: perhaps "marine" would be more suitable here, as more general and inclusive. in this case, I would suggest the following structure: ...including ecosystem complexity and diﬃculty of sampling the marine realm.

**Answer**: The issue has been addressed in accordance with the reviewer’s suggestion.

### +Line 57 “Ecological Niche”: why are these capitalized?

**Answer**: The issue has been addressed in accordance with the reviewer’s suggestion

### +Line 60 “develop”: developed

**Answer**: The issue has been addressed in accordance with the reviewer’s suggestion

### +Line 76 “introduction”: this section could beneﬁt from some more references regarding SDMs and more. suggested refs:

### <https://www.frontiersin.org/journals/marine-science/articles/10.3389/fmars.2017.00421/full>

### <https://nsojournals.onlinelibrary.wiley.com/doi/full/10.1002/ecog.07702>

### <https://onlinelibrary.wiley.com/doi/full/10.1111/gcb.16371>

**Answer**: we added the following paragraph to the introduction after 3^rd^ paragraph:

SDMs have become central to marine biogeography because they allow inference of species–environment relationships and prediction of distributions where observations are sparse. A systematic review of 236 marine SDM studies showed that SDMs are now widely applied but that robust practice requires transparent data handling, consistent evaluation metrics, and explicit reporting of uncertainty (Robinson et al., 2017). Recent syntheses highlight major methodological advances, including increased use of machine-learning algorithms, three-dimensional and depth-explicit modelling, and improved handling of dynamic oceanographic variability, while also noting persistent biases in occurrence data and challenges linked to the spatial–temporal dynamics of the ocean (Klaassen et al., 2025). When SDMs are projected under future climate scenarios, structural model differences and extrapolation into novel environmental conditions contribute substantial uncertainty, which can exceed Earth system model uncertainty at multi-decadal to century scales, underscoring the need to quantify uncertainty when such projections are used for management or climate-risk assessments (Brodie et al., 2022). Together, these developments establish the current expectations for marine SDMs and provide the context in which next-generation frameworks such as AquaX have been developed.

### +line 79 “SDG”: please spell out the full word for this ﬁrst mention

**Answer**: The issue has been addressed in accordance with the reviewer’s suggestion

### +line 128 “also strengthens”: suggestion: would also strengthen

**Answer**: The issue has been addressed in accordance with the reviewer’s suggestion

### +line 129 “makes”: make

**Answer**: The issue has been addressed in accordance with the reviewer’s suggestion

### +line 132: delete “and”; comma aeer “algorithms

**Answer**: The issue has been addressed in accordance with the reviewer’s suggestion

### +line 143 “enable”: enables

**Answer**: The issue has been addressed in accordance with the reviewer’s suggestion

### +line 148 “evidences”: evidence

**Answer**: The issue has been addressed in accordance with the reviewer’s suggestion

### +line 151 “explore”: is it to explore or really to present?

**Answer**: thank you for pointing it out, we replaced ‘explore’ with ‘present’

### +Line 165 “AphiaID”: please add this “Aphia unique identiﬁers” since this is the ﬁrst mention

**Answer**: The issue has been addressed in accordance with the reviewer’s suggestion

### +Line 195 “GOODOCC”: I don't think this has been previously deﬁned in the text

**Answer**: As we never mentioned the column anywhere in manuscript, we decided to remove the name of the column. Sentence is re-written as following: Other original OBIS flags, such as missing depth information or occurrences falling outside known bathymetric limits, were retained in original flag column but not automatically considered erroneous, and their value was left blank in standardized binary flag column.

### +Line 213 “FishBase”: add “for ﬁshes”

**Answer**: The issue has been addressed in accordance with the reviewer’s suggestion

### +Line 214 “SeaLifeBase”: add “for other marine organisms”

**Answer**: The issue has been addressed in accordance with the reviewer’s suggestion

### +Line 217 “describe precisely”: precisely describe

**Answer**: The issue has been addressed in accordance with the reviewer’s suggestion

### +Line 240 “varies here from”: varies based on? or diﬀers from? which of the two?

**Answer**: thank you for pointing it out, we re-placed it to “differs from”

### +Line 278 “buﬀering of 10o of longitude and latitude”: isn't this huge? Ten degrees of latitude ≈ 1,110 km. so even for 100 years, this would be around 5 degrees change. also, a poleward shift is by deﬁnition directional, while buﬀering expands in all directions.

**Answer**: We agree that a 10° buffer appears large in absolute distance. The buffer size was selected to approximate the mean poleward shift of marine species reported by Lenoir et al. (2020), who found an average movement of 5.9 ± 0.9 km yr⁻¹ over recent decades. Projecting to 2100 requires covering roughly a century of potential redistribution, which corresponds to approximately 5° of latitude under this rate. The expanded 10° threshold was therefore chosen to ensure full spatial coverage for species with above-average or non-linear responses to warming.

In addition, several species have Expert Range Maps (ERM) that are outdated or constrained relative to their currently observed distributions; for these cases, even a 10° buffer can fail to encompass known occurrences (e.g., Mugil cephalus, Ammodytes hexapterus). The buffer is thus not intended to represent a predicted shift directionally, but rather to define a modelling domain that prevents artefactual projections into the wrong ocean basin or hemisphere. Although many species shift poleward, others exhibit depthward or multidirectional changes, and robust shift estimates do not exist for the ~32,000 species modelled across all taxa. For fishes, empirical rates are comparatively well characterized, but this is not the case for many invertebrate groups that comprise more than half of the modelled species.

Finally, we note that this buffering approach is a pragmatic interim solution. Ongoing work in our group is focused on refining dynamic ranges using connectivity-informed constraints, which will allow more mechanistic spatial limits in future AquaMaps iterations.

### +Line 368 “(2000-2010) and two future periods (2050-”: what about 2011-2049?

**Answer**: Bio-Oracle v3 provides environmental variables aggregated by decade. In this study, we focused on three periods—present, mid-century, and end-century. The present is represented by conditions from the 2000s, mid-century by the 2050s, and end-century by the 2090s. This temporal selection is arbitrary; additional decades could be modelled, but producing projections for these three periods required approximately one year of continuous computation, making further periods currently impractical.

### +Line 413 “unique environments”: what is the purpose of this process?

**Answer**: The purpose of defining “unique environments” was to reduce sampling bias by ensuring that each species was represented across the full range of environmental conditions it occupies, rather than by the spatial density of occurrence records. Because SDMs infer habitat suitability from environmental gradients, this step prevents over-representation of well-sampled regions and avoids removing ecologically informative points solely due to geographic proximity.

### +Line 444 “models algorithm”: Model algorithms?

**Answer**: The issue has been addressed in accordance with the reviewer’s suggestion

### +Line 508-9 “three environmental parameters available at the time”: which ones? it would be useful to mention in a parenthesis

**Answer**: three environmental variables are depth, sea-surface temperature, and ice edge association. We mentioned them in parathesis as suggested.

### +Line 515 “through”: the new AquaX framework

**Answer**: The issue has been addressed in accordance with the reviewer’s suggestion

### +Line 523 “replicable on”: in

**Answer**: The issue has been addressed in accordance with the reviewer’s suggestion

### +Line 537-40: add one or two relevant references

**Answer**: The importance of enhanced resolution in marine SDMs is significant, particularly in modeling smaller-scale features such as MPAs and coastal zones. Traditional coarse-resolution models often fail to accurately capture critical habitat details that are essential for effective conservation planning (Raymaekers et al., 2016). So we added the following reference:

Raymaekers D, Knaeps E, Van De Kerchove R. Remote Sensing for a Sustainable Coastal Management. Qatar Foundation Annual Research Conference Proceedings Volume 2016 Issue 1. Qatar National Convention Center (QNCC), Doha, Qatar,: Hamad bin Khalifa University Press (HBKU Press); 2016. doi:10.5339/qfarc.2016.EEPP1662

### +Line 550 “frame”: frames?

**Answer**: The issue has been addressed in accordance with the reviewer’s suggestion

### +Line 554: in “the” FISH-MIP and CMIP projects

**Answer**: The issue has been addressed in accordance with the reviewer’s suggestion

### +Line 585: A tool for expert-based spatial delineation of species ranges?

**Answer**: The issue has been addressed in accordance with the reviewer’s suggestion

### +Line 588: perhaps better “AquaMaps” add something along the lines of ", the basis of AquaX,"

**Answer**: The issue has been addressed in accordance with the reviewer’s suggestion

### +Line 596-600: relevant references that express such needs could be cited here

**Answer:** Evidence underscores that adapting to climate change involves not merely recreational or economic considerations but also the preservation of biodiversity and ecosystem services that these marine environments provide. As outlined by Fuchs et al., the link between climate adaptation and marine conservation is crucial for formulating effective management strategies that consider both ecological integrity and human livelihoods amidst growing environmental pressures.While Halpern et al, highlighted effective marine spatial planning must consider ecological, economic, and social objectives while adapting to conditions driven by climate change. We added the following references:
Fuchs G, Kroos F, Scherer C, Seifert M, Stelljes N. Exploring marine conservation and climate adaptation synergies and strategies in European seas as an emerging nexus: a review. Front Mar Sci. 2025;12: 1542705. doi:10.3389/fmars.2025.1542705

Halpern BS, Klein CJ, Brown CJ, Beger M, Grantham HS, Mangubhai S, et al. Achieving the triple bottom line in the face of inherent trade-offs among social equity, economic return, and conservation. Proc Natl Acad Sci USA. 2013;110: 6229–6234. doi:10.1073/pnas.1217689110

### +Line 609 “to continue to”: add “further”

**Answer**: The issue has been addressed in accordance with the reviewer’s suggestion

### +Line 614 “actions”: reference? most statements in this part could be supported by relevant refs. this section is still discussion, not just results

**Answer**: Thank you for highlighting this issue. We have added appropriate references to substantiate the claims in this paragraph.

### +Line 619 “analysis”: analyses

**Answer**: The issue has been addressed in accordance with the reviewer’s suggestion

### +Line 624 “The new”: it would be more intuitive if you presented the results in this section ﬁrst and then have the discussion part, which is the ﬁrst paragraph, to my understanding

**Answer**: The introductory paragraph has been relocated to the end of the section, ensuring that results are presented first, followed by a concluding discussion highlighting the significance of the work.

### +Line 624-5 “which presented with diﬀerent ecology”: this phrasing is confusing

**Answer**: We defined ‘different ecologies’ as benthic, pelagic or neritic species. In the prior AquaMaps version, all species were modelled using the same surface-layer environmental conditions and methodology, whereas our approach explicitly distinguishes among ecological guilds and tailors environmental layers and modelling protocols to each group.

We added clarification to the sentence: “The new AquaX framework was run for all species with sufficient occurrence data, which presented with different ecology(i.e. benthic vs pelagic vs pelagic-neritic), allowing to unveil the advantage of the methodology especially for coastal species.”

### +Line 626 “the outputs”: delete “the”

**Answer**: The issue has been addressed in accordance with the reviewer’s suggestion

### +Line 628: “the” ERM

**Answer**: The issue has been addressed in accordance with the reviewer’s suggestion

### +Line 629 “extended”: “extends”

**Answer**: The issue has been addressed in accordance with the reviewer’s suggestion

### +Line 635 “habitat modiﬁcations”: what is meant by that?

**Answer**: thank you for pointing it out, here we meant other environmental variables. The error has been corrected

### +Line 640 “RCP”: was this deﬁned before in the text?

**Answer**: spelled in full here

### +Line 642-4: references for this statement?

**Answer**: this is the reported result of the study, here we propose/speculate on potential application of the demonstrated result.

### +Line 646 “species”: reference for this statement?

**Answer**: this is the reported result of the study, here we propose/speculate on potential application of the demonstrated result.

### +Line 648 “For example”: suggested: For example, in the case of sand trout, an increase in HSI is projected in its northward range by mid-century (Fig. 3E–F), along with a potential shift to deeper waters (blue areas below the red strip in Fig. 3F).

**Answer**: The issue has been addressed in accordance with the reviewer’s suggestion

### +Line 649 “mid of the century”: mid-century

**Answer**: The issue has been addressed in accordance with the reviewer’s suggestion

### +Line 654 “compute to”: should this be deleted?

Answer: “to” was not needed, it should be and computer climate driven indices (e.g., local extinctions, invasions). Te typo has been fixed.

### +Line 658 “providing”: this sentence is not ﬁnished

**Answer**: Thank you for spotting it! Indeed, the sentence been cut off. The full sentence should read as follows: By analyzing these binary maps over time (Fig 4), we can compute the spatial extent of habitat loss and gain, providing not only insights into habitat degradation or improvement but also into actual species distribution dynamics for policy makers and stakeholders.

### +Line 666: Furthermore, the AquaX-derived species richness …

**Answer**: The issue has been addressed in accordance with the reviewer’s suggestion

### +Line 668 “resampled”: resampling?

**Answer**: The issue has been addressed in accordance with the reviewer’s suggestion

### +Line 669 “using”: replace with “with” or “employing”

**Answer**: The issue has been addressed in accordance with the reviewer’s suggestion

### +Line 670 “dataset”: datasets

**Answer**: The issue has been addressed in accordance with the reviewer’s suggestion

### +Line 687 “approach”: I would delete this

**Answer**: The issue has been addressed in accordance with the reviewer’s suggestion

### +Line 706 “depth ranges”: I wonder if depth data from surveys have been or can be integrated. suggested source to explore: https://james-thorson.github.io

**Answer**: We appreciate the suggestion and agree that integrating depth information from survey-based datasets would be valuable. We are keen to incorporate depth ranges from all reliable sources, including structured survey products such as those developed by Thorson and colleagues. At the same time, our workflow relies on reproducible and persistently maintained data streams. For this reason, we currently prioritise data sources with stable, curated infrastructures—such as SeaLifeBase, FishBase, and WoRMS—which are widely used, continuously updated, and maintain long-term versioning. We would welcome the opportunity to integrate additional survey-derived depth information, but a key challenge is ensuring that such datasets remain traceable and persist through time. When AquaX is updated in future cycles (e.g., in four years), external datasets may have been modified, relocated, or removed, which could compromise reproducibility or prevent re-building the database. Sustained, curated repositories such as SeaLifeBase, FishBase, and WoRMS therefore remain essential.For this reason, when we work with collaborators or receive requests to incorporate new depth information, we encourage contributors to submit their data directly to FishBase, SeaLifeBase, or WoRMS. Centralised submission to these platforms ensures that the information is curated, up to date, and accessible in a single stable repository, which in turn allows AquaMaps and AquaX to integrate these improvements reliably in future updates.

### +Line 720 “with a depth range from 0-12m”: based on what?

**Answer**: depth range taken from FishBase.

### +Line 747: records, with most …

**Answer**: The issue has been addressed in accordance with the reviewer’s suggestion

### +Line 772: veriﬁcations), and 5.4%

**Answer**: The issue has been addressed in accordance with the reviewer’s suggestion

### Line 805: any relevant refs?

Answer: we searched for reference in the oceanic realm and unfortunately could not find one. There are similar attempt done on the terrestrial but with different constrains and biases. Therefore we did not include terrestrial reference to this point.

### +Line 807-8 “and attempt to follow update on ERM at a by-annually level”: this sentence needs rephrasing

**Answer**: Indeed sentence is confusing. We re-wrote tit in the following way: “In our framework, we chose rely on expert opinion and use ERM when available; while we recognize that ERMs are not ideal, we plan to refine and improve them in the next iteration of the model.”

### +Line 834: high-sea

**Answer**: The issue has been addressed in accordance with the reviewer’s suggestion

### Line 857-9: so, it aﬀects the SSP projections here as well which is quite important

**Answer**: we apologize that the text was confusing , we have revise this sentence accordingly to the reviewer comment.

### +Line 862: this seems to be addressed in a previous section - why not combine the two? a couple of references would be useful for readers here

**Answer**: thank you for the suggestion, the sections has been merged together, we added references to support the claims in this section.

### +Line 872 “unabling”: hindering .. from making?

**Answer**: The issue has been addressed in accordance with the reviewer’s suggestion

### +Line 908 “disk”: was this explained previously? perhaps a short explanation would still help here

**Answer**: Thank you for suggestion, we added the description of disk as “a spatially explicit data partitioning option implemented in biomod2, where absences (and/or pseudo-absences) are excluded within a specified radius around presences,” and cited Thuiller et al.

### +Line 921 “bottom trawling”: ﬁshing was included in this paper, perhaps it could be cited

### https://[www.pnas.org/action/oidcCallback?idpCode=connect&error=login_required&error_description=](http://www.pnas.org/ac2on/oidcCallback?idpCode=connect&error=login_required&error_descrip2on) Login+required&state=Dps2IO0LOrpSUAYYguc7KjWtugvQmVzet9SRzoptjvlX2D8yKiJIuCAF5rn25evTKSYeBJ HZxgNLEKhORjMUMTOXbOnAuqUcDJMGMt4%2BX7v0loW6TTUMWc%2BXGrsT9EEsIXiBtzIJTsTd4F0rE1T m6w%3D%3D

**Answer**: We were unable to confirm the exact paper from the provided login-required link.But we added the following citations to support the claim that anthropogenic activities like bottom trawling, coastal development, and marine pollution significantly influence these ecosystems and their inhabitants.:

Holon F, Mouquet N, Boissery P, Bouchoucha M, Delaruelle G, Tribot A-S, et al. Fine-Scale Cartography of Human Impacts along French Mediterranean Coasts: A Relevant Map for the Management of Marine Ecosystems. Bianchi CN, editor. PLoS ONE. 2015;10: e0135473. doi:10.1371/journal.pone.0135473

Álvarez-Romero JG, Devlin M, Da Silva ET, Petus C, Ban N, Pressey RL, et al. A novel approach to model exposure of coastal-marine ecosystems to riverine flood plumes based on remote sensing techniques. MarXiv; 2018. doi:10.31230/osf.io/kh7aw

Tuholske C, Halpern BS, Blasco G, Villasenor JC, Frazier M, Caylor K. Mapping global inputs and impacts from of human sewage in coastal ecosystems. Veettil BK, editor. PLoS ONE. 2021;16: e0258898. doi:10.1371/journal.pone.0258898

### +line 928: a reference or two?

**Answer**: we added the following references:

Hattab T, Albouy C, Lasram FBR, Somot S, Le Loc’h F, Leprieur F. Towards a better understanding of potential impacts of climate change on marine species distribution: a multiscale modelling approach. Global Ecology and Biogeography. 2014;23: 1417–1429. doi:10.1111/geb.12217

Becker EA, Forney KA, Redfern JV, Barlow J, Jacox MG, Roberts JJ, et al. Predicting cetacean abundance and distribution in a changing climate. Beger M, editor. Diversity and Distributions. 2019;25: 626–643. doi:10.1111/ddi.12867

Hattab et al. highlighted that the combined effects of climate change and habitat loss could lead to drastic consequences for marine ecosystems, underscoring the need for integrating these factors into species distribution models (SDMs) for robust conservation management decisions. Becker et al. emphasize the limitations of predictive models in marine spatial planning when habitat loss is excluded. The authors argue that marine protected areas (MPAs) and related mitigation strategies may not perform effectively if they fail to account for the risks imposed by habitat destruction. Thus, only by incorporating habitat dynamics into projections can we develop meaningful interventions that enhance marine biodiversity resilience.

### +Line 1228: which color represents what (invasion vs extinction)? there is no color scale

**Answer**: thank you for spotting the error, the image has been cropped in the manuscript, obscuring the legend. However, the pdf of the image we shared has the full legend attached:

### +Line 1229: the middle … and end … of the century

**Answer**: The issue has been addressed in accordance with the reviewer’s suggestion

### +Line 1233-4 “lost or gained”: which color represents which?

**Answer**: thank you for spotting the error, the image has been cropped in the manuscript, obscuring the legend. However, the pdf of the image we shared has the full legend attached.

### +Line 1270: my comments on this Fig. are resolved when reading the text, but I believe these details should be added to the ﬁg. legend for clarity

**Answer**: addressed in the comment above

### +Line 1272 “ﬂags” and “occurrence type”: what kind of ﬂags? And consider adding two examples. (e.g., xxx and xxx).

**Answer**:

There is no single standardized set of flag examples because two flag columns are used: the original data-source flags (original_flags) and the standardized binary flags (GOODOCC). The content of original_flags depends on the provider. For example, GBIF does not supply native flags, so this column remains empty, as shown in the diagram. In contrast, OBIS provides extensive data-quality flags (see OBIS Manual: “18 Data quality flags”, [18 Data quality flags | The OBIS Manual](https://manual.obis.org/dataquality.html)), and these are recorded in original_flags. As indicated in the diagram, occurrences are assigned a standardized flag value of 0 only when the original OBIS flags indicate that the point falls on land, has coordinates of (0,0), or contains other latitude/longitude errors. AquaMaps uses a binary flagging system in which 1 indicates a good occurrence and 0 indicates a bad occurrence, as described in the manuscript. For this reason, all flags are standardized to a binary format and stored in the GOODOCC column. The data-cleaning steps (ie assigning flags) then continue as outlined in subsequent diagrams. For occurrence type: we have added the examples : “(e.g, human observation, Machine observation, fossil specimen , museum collection).”

For the Figure caption, we re-wrote the description the following way:

**S1 Fig1. Conceptual diagram of the process of compiling and standardizing AquaMaps, OBIS and GBIF occurrence data.** Abbreviations used: OCC – occurrence data, original_flags – column in OCC containing the original data-source flags (if available), GOODOCC – column in OCC containing standardized binary flags, basisOfRecord – column in OCC containing occurrence/observation type (e.g., human observation, machine observation, fossil specimen, museum collection).

### +Line 1287 “ CSI, AUC and TSS”: these should be deﬁned in the legend so it is stand-alone

**Answer**: abbreviations spelled in full: “Critical Score Index (CSI), Area Under the Curve (AUC), and True Skill Statistic (TSS) are reported for 300 model runs”.

### +Line 1288 “300 runs. Boxplot for each single models are shown as well as outliers (point).”: please rephrase for better ﬂow and typos

**Answer**: re-written caption as following: Fig. S6: Validation metrics of for *Thunnus obesus* (Lowe, 1839) across all models. Critical Score Index (CSI), Area Under the Curve (AUC), and True Skill Statistic (TSS) are reported for 300 model runs. Boxplots summarize the distribution of each metric for each model, with individual points representing outliers.

### +Line 1289: what does this mean "DepthRangeShallow_NA"? that there are 6000 missing values of what? of shallow species? the NA at the end is also confusing

**Answer**: “DepthRangeShallow_NA” indicates records where the shallow-depth value is missing in the metadata. It does not mean “shallow species.” It simply shows that the variable DepthRangeShallow contains NA (missing) for those observations. The label ends in “_NA” because the UpSet plot software automatically appends “_NA” to denote missingness in that specific variable.

To clarify this, we added this sentence to Fig S6 and S7 captions: “Abbreviations: HAB- habitat type, DepthRangeDeep(Shallow)- max(min) depth of the species distribution range. Variable labels ending in “_NA” (e.g., “DepthRangeShallow_NA”) indicate that the corresponding variable contains a missing (NA) entry; this notation is automatically assigned by the plotting function and does not refer to biological characteristics of the species.”

### +Line 1301-2: italicize the species

**Answer**: The issue has been addressed in accordance with the reviewer’s suggestion

### +Fig. S6: x axis label typo “validation”

**Answer**: thank you for spotting the typo, it has been fixed
